# High dissolved organic carbon release by benthic cyanobacterial mats in a Caribbean reef ecosystem

**DOI:** 10.1038/srep08852

**Published:** 2015-03-09

**Authors:** Hannah J. Brocke, Frank Wenzhoefer, Dirk de Beer, Benjamin Mueller, Fleur C. van Duyl, Maggy M. Nugues

**Affiliations:** 1 Max Planck Institute for Marine Microbiology (MPI Bremen), Celsiusstr. 1, 28359 Bremen, Germany; 2 Leibniz Center for Tropical Marine Ecology (ZMT), Fahrenheitstr. 6, 28359 Bremen, Germany; 3 CRIOBE – USR 3278, CNRS-EPHE-UPVD, 58 Avenue Paul Alduy, 66860 Perpignan Cedex, France; 4 Alfred Wegener Institute, Am Handelshafen 12, 27570 Bremerhaven, Germany; 5 Royal Netherlands Institute for Sea Research (NIOZ), P.O. Box 59, 1790AB Den Burg, Texel, The Netherlands; 6 CARMABI Foundation, Piscaderabaai z/n, P.O. Box 2090, Willemstad, Curaçao; 7 Laboratoire d'Excellence “CORAIL”

## Abstract

Benthic cyanobacterial mats (BCMs) are increasing in abundance on coral reefs worldwide. However, their impacts on biogeochemical cycling in the surrounding water and sediment are virtually unknown. By measuring chemical fluxes in benthic chambers placed over sediment covered by BCMs and sediment with BCMs removed on coral reefs in Curaçao, Southern Caribbean, we found that sediment covered by BCMs released 1.4 and 3.5 mmol C m^−2^ h^−1^ of dissolved organic carbon (DOC) during day and night, respectively. Conversely, sediment with BCMs removed took up DOC, with day and night uptake rates of 0.9 and 0.6 mmol C m^−2^ h^−1^. DOC release by BCMs was higher than reported rates for benthic algae (turf and macroalgae) and was estimated to represent 79% of the total DOC released over a 24 h diel cycle at our study site. The high nocturnal release of DOC by BCMs is most likely the result of anaerobic metabolism and degradation processes, as shown by high respiration rates at the mat surface during nighttime. We conclude that BCMs are significant sources of DOC. Their increased abundance on coral reefs will lead to increased DOC release into the water column, which is likely to have negative implications for reef health.

Cyanobacteria are a common benthic and planktonic component of coral reef ecosystems^1^. They are important contributors to primary production, nitrogen fixation and reef building^1^,^2^. In recent decades however, many coral reefs have experienced massive blooms of noxious benthic species forming dense mats over the seabed^3^,^4^,^5^,^6^. These benthic cyanobacterial mats (BCMs) inhibit coral settlement and recruitment, potentially limiting the ability of corals to recover from disturbances^7^. Some cyanobacteria also act as coral pathogens^8^ and disturb coral reef-associated microbial communities^9^. The blooms are difficult to control by grazers as these organisms produce potent allelochemicals that deter feeding^10^,^11^. They also appear to be facilitated by environmental conditions associated with anthropogenic impacts and global climate change, which are likely to become worse in the near future. Therefore, it is predicted that their abundance will increase in the coming decades^2^,^12^.

Aquatic primary producers, such as cyanobacteria, release part of their photosynthetically fixed carbon as DOC into the water column e.g. Refs. 13,14,15,16. Therefore, changes in the abundance of primary producers can alter the quantity and chemical composition of organic materials supplied to the reef environment and have long-term impacts on reef communities^17^,^18^,^19^. For example, macroalgal exudates are thought to play a pivotal role in community shifts from coral to algal dominance occurring on many coral reefs worldwide^18^. Corals can retain organic materials by trapping particles from the water column, which are subsequently remineralized^20^ and they can release DOC^16^,^21^,^22^. However, benthic macroalgae release higher amounts of, and comparatively more neutral sugar rich, DOC than corals^19^,^23^. Macroalgal exudates have been shown to induce microbe-induced coral mortality^24^, foster faster growth of less diverse and more pathogenic microbes than coral exudates^23^ and favor net heterotrophic metabolism^19^.

Despite the increasing abundance of BCMs and the recent focus on biogeochemical cycling and microbial processes in coral ecosystems, hardly anything is known about the DOC release of BCMs and their impact on carbon cycling in coral reefs. BCMs release DOC as photosynthates^14^ during the day and as products of anaerobic metabolism and degradation processes at night^15^. Their exudates are thought to play an important role in controlling bacterioplankton activity in aquatic systems^25^. However, most studies on carbon cycling in coral reefs have focused on planktonic cyanobacteria^26^. The goal of this study was to investigate the influence of BCMs covering large areas of coral reef sediment on the dissolved carbon flux in Curaçao, Southern Caribbean. We (1) determined DOC, dissolved inorganic carbon (DIC), oxygen and inorganic nutrient fluxes over diel cycles using benthic chambers placed over sediment covered by BCMs and sediment with BCMs removed, (2) assessed the influence of BCMs on sedimentary carbon cycling by comparing the carbon budgets of both experimental treatments, and (3) estimated the contribution of BCMs to the DOC pool at the reef scale by assessing the cover of the major benthic components at our study site and their respective DOC release rates over a diel cycle using the results of this and other studies. Finally, the vertical distribution of oxygen was determined across the sediment-water interface with and without the presence of BCMs using microsensors to investigate photosynthetic and respiration processes.

## Results

Fluxes of O_2_, DIC and DOC in sediment covered with BCMs (BCM treatment) were significantly higher than in sediment with BCMs experimentally removed (CTRL 1 treatment) (Table 1; posthoc Scheffé tests, *P* < 0.05). During the day, sediment covered with BCMs released O_2_ and took up DIC, with 5–6 times higher fluxes than sediment with BCMs removed. During the night, O_2_ was respired and DIC was released, with 3–4 times higher fluxes. Sediment covered with BCMs net released 1.4 (±1.2 SD) mmol C m^−2^ h^−1^ DOC during the day and doubled this amount during the night [3.5 (±2.0) mmol C m^−2^ h^−1^]. Conversely, sediment from which BCMs were removed on average took up DOC during both day and night [i.e. −0.9 (±0.6) and −0.6 (±0.7) mmol C m^−2^ h^−1^]. Concentrations of inorganic nutrients over the sediment water interface were below or close to detection limits in all treatments (NH_4_^+^ < 0.07 μM, NO_2_^−^/NO_3_^−^ < 0.05 μM, PO_4_^3−^ < 0.01 μM). Therefore, fluxes were not estimated. However, the uniformly low concentrations are indicative of low nutrient fluxes and suggest that cells from the BCMs were not lysing/dying during the incubations.

Benthic chambers were also placed over undisturbed sediment without BCMs (CTRL 2 treatment). This second control did not differ from the first control (sediment from which were BCMs removed) in either oxygen or DIC fluxes (Table 1; posthoc Scheffé tests, *P* > 0.05). DOC fluxes were not measured on naturally bare sediments. During the incubations, salinity was consistently 35 PSU in all chambers. Daytime photosynthetic active radiation did not differ among the experimental treatments (Table 1; 1-way ANOVA, *P* > 0.05). Water temperatures were slightly lower during the incubations on naturally bare sediments compared to the other treatments, but differences were minute (≤0.4°C) (Table 1).

**Figure 1:**
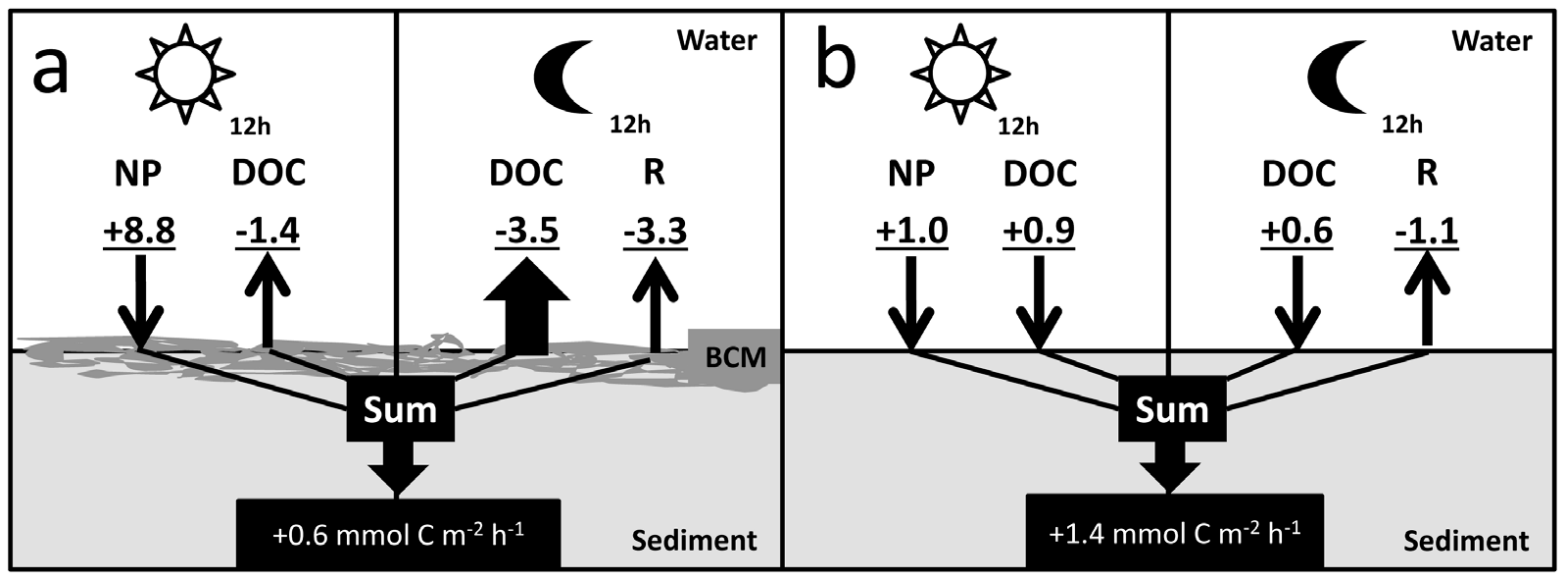
Day and night carbon budgets for sediment with BCMs (a) and without BCMs (b). (+) indicates uptake and (−) loss of carbon from sediment. NP = Net production (day); R = respiration (night).

Over a 24 h diel cycle, the carbon budgets indicated that the presence of BCMs reduced the net DOC input into the sediment by 57%, with net uptake rates of 0.6 (±2.8) mmol C m^−2^ h^−1^ for sediment with BCMs and 1.4 (±1.5) mmol C m^−2^ h^−1^ for sediment with BCMs removed (Fig. 1). Rates of daytime DOC release by BCMs were within the range of rates reported for macroalgae or turf, both of which generally also released DOC (Table 2). No clear trends were recognizable among macroalgal divisions when pooling the different studies. Corals generally showed a net uptake of DOC. Nighttime DOC release by BCMs was higher than rates obtained in *ex situ* dark incubations for most primary producers in coral reefs.

At our study site, the ecosystem compartments that produced DOC (BCMs, macroalgae and turf) covered 24, 17 and 19% of the seabed, respectively (Table 3a). Averaged over the reef and over a 24 h cycle, the BCMs, macroalgae and turf were estimated to release DOC at rates of 0.59, 0.04 and 0.11 mmol C m^−2^ reef h^−1^, respectively. The two other ecosystem compartments (corals and bare sediments) did not release DOC over a 24 h cycle. Thus, BCMs contributed to 79% of the total DOC released. Taking into account the net uptake of DOC by corals (13% cover) and bare sediments (25% cover), the reef yielded a net release of DOC (+0.19 mmol C m^−2^ reef h^−1^). In a theoretical scenario with all BCMs removed, the reef yielded a net uptake of DOC (−0.6 mmol C m^−2^ reef h^−1^) (Table 3b). Based on our model, the reef would switch from a net sink to a net source of DOC at 19% BCM coverage.

**Figure 2:**
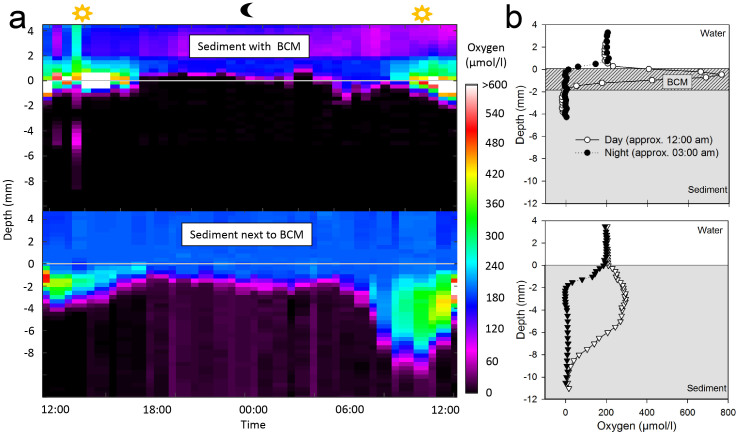
*In situ* oxygen profiles. (a) over a period of 24 hours in sediment covered with BCMs and next to BCMs. Colors indicate oxygen concentration over time and depth. (b) Examples of *in situ* O_2_ profile at the sediment-water interface during day and night with BCMs and next to BCMs.

O_2_ microprofiles measured over a 24 h cycle showed that, during the day, maximal O_2_ concentrations were 2–8 times higher in sediment covered with BCMs than in sediment next to BCMs (Fig. 2). The depth of the oxygenated layer was reduced when BCMs covered the sediment. During the night, the sediment and BCMs became rapidly anoxic up to the surface.

## Discussion

The results of this study provide the first rates of DOC release by BCMs on coral reefs. Our comparison between daytime and nighttime rates with previously reported DOC releases by macroalgae, turfs and corals on coral reefs worldwide suggest that BCMs release comparatively high quantities of DOC into the water column, especially at night. DOC release by benthic primary producers on coral reefs has been shown to be positively related to light intensity^16^,^22^,^27^,^28^. Similarly, DOC release by hot spring cyanobacterial mats is enhanced under elevated light intensities^14^. Therefore we expected nocturnal DOC release to be lower than during the day. During daytime, DOC from BCMs is most likely released by the excretion of photosynthates, as supported by the high rates of oxygen production. During nighttime, the released DOC most likely consists of products from incomplete organic matter degradation and fermentation^29^, as supported by the high heterotrophic activities in the mat. Several species of *Oscillatoria* maintain their metabolism by glycogen-glucose fermentation to survive and grow under dark and anaerobic conditions^30^,^31^,^32^.

When roughly estimating the areal DOC release on the reef flat at Pest Bay by combining literature data and results of this study, BCMs, with a coverage of 24%, provided the largest positive contribution to the DOC pool. The presence of BCMs also annihilated the capacity of sediment to act as a net sink for DOC. This largely affected the DOC pool at reef scale, with the reef switching from being a net sink to a net source of DOC when BCMs covered more than 19% of the seabed. Unlike other benthic primary producers on coral reefs, BCMs released large amounts of DOC during both day and night. DOC released by primary producers, such as algae, corals and phytoplankton, is the result of the release of excessive photosynthates during the day and linearly increases with light intensity until a light maximum threshold is reached and DOC release becomes constant or decreases^22^,^27^,^33^. Thus their DOC releases are typically close to zero below a depth of 20 m^16^,^22^,^27^,^34^. For BCMs, the same declining trend is likely to occur for their DOC release during the day^14^; however, their dark DOC release could occur over a larger depth gradient. The release of fermentation products during nighttime is dependent on productivity and the built up of glycogen^35^. Cyanobacteria use large pigment-protein complexes called phycobilisomes which capture photons between the blue and red regions of the spectrum that are not efficiently trapped by chlorophyll^36^. Since these shorter wave lengths predominate at deeper depths, BCMs could maintain their productivity across a large depth gradient due to light adaptation.

The presence of BCMs reduced the net carbon gain in the sediment by more than half. Sediment with BCMs showed a net primary production, high respiration rates and released large quantities of DOC. In contrast, sediment with BCMs removed revealed a lower net production and also respired less and took up DOC from the water column. Sediments, including carbonate sediments on coral reefs, are a well-known sink for organic matter, such as DOC, through mineralization and burial^37^,^38^,^39^,^40^,^41^. For example, Werner et al.^39^ estimated that the total area of Heron Reef occupied by sediments (sediment area = 19.5 km^2^) showed annual turnover rates of 3 700 to 13 000 t C. Our results suggest that the presence of BCMs over coral reef sediment may influence sedimentary recycling processes and result in larger DOC pools in the water column.

The impact of the released DOC from BCMs into the surrounding water will depend on its bioavailability. Lactate, glycolate, formate, ethanol and acetate are released during nocturnal fermentation processes in the genus *Oscillatoria*^32^. Such compounds are easily degraded by microbes. They could favor more heterotrophic metabolism, as shown for algal exudates^19^. Kelly et al.^42^ documented a 10 fold higher heterotrophic metabolism above cyanobacterial/algal dominated reefs called Black Reefs in the Central Pacific. This could lead to a system-wide decrease in DOC concentrations via enhanced heterotrophy and co-metabolism of refractory carbon that occurs when microbes are given an excess labile carbon^43^. The released DOC could also indirectly affect nearby corals by enhancing microbial growth and respiration, in particularly that of opportunistic pathogens^18^. Kelly et al.^42^ found an increase in virulence genes and known pathogens on black reef sites and demonstrated that corals were killed by black reef rubble through microbial activity in microcosm experiments. Furthermore, BCMs produce potent allelochemicals^10^,^11^,^44^. Both lipo- and hydrophilic extracts from two species of *Lyngbya* cyanobacteria enhanced the growth of coral reef-associated bacterial taxa^9^.

There are some uncertainties in our budget calculation. Firstly, in our reef-scale DOC calculations, we used DOC fluxes from sediment with BCMs experimentally removed, as no DOC data were available for undisturbed BCM-free sediment. As all other fluxes were the same, we assume that the DOC fluxes were representative for a natural situation. Both undisturbed sediment and sediment with BCMs experimentally removed had low oxygen fluxes, suggesting low productivity and similar DOC release rates. Secondly, our budget calculation over whole reefs includes literature data obtained from ex situ incubations (fluxes on corals, turf and macrophytes). Stressfull sampling and maintenance in the artificial laboratory environment can lead to overestimation of the DOC release, due to cutting of tissue and unrealistic hydrodynamic conditions^16^,^34^. Indeed we observed a rapid deterioration of the health of the mats upon ex situ incubations (lysis), thus for the BCMs we relied on our in situ data. In short, the literature data may provide an overestimation of the DOC release by corals and turf, which further emphasizes the importance of BCMs for carbon cycling in coral reefs. Lastly, the estimated DOC fluxes were highly variable. Potential cause for this variability includes variation in mat age and density and environmental parameters such as light, temperature and advection. However, if the average day and night DOC fluxes are lowered by one standard deviation (i.e. encompassing 68% of the mats in a normal distribution), BCMs still represent 56% of the total DOC released over a 24 h diel cycle at our study site, suggesting that our conclusions are relatively robust.

Although further investigations of DOC release by BCMs and undisturbed sediment are warranted, this study supports that BCMs are significant sources of DOC and can strongly contribute to the DOC pool on coral reefs. Their increased abundance will lead to increased DOC supply to the reef overlying water and have profound consequences for element cycling, microbial processes and coral survival in tropical reefs.

## Methods

### Study site

The experiments were performed between September and November 2011 at 7 to 8 m water depth on a fringing coral reef at Pest Bay on the leeward side of the island of Curaçao (Fig. S1a; 12°09′894″N 69°00′657″W). At this depth, the reef consisted of coral heads separated by sand patches largely covered by brown-colored BCMs (Fig. S1b). The mats were primarily dominated by *Oscillatoria bonnemaisonii*, a common bloom-forming genus on coral reefs^45^.

### *In situ* benthic chamber experiment

To investigate the exchange rates of O_2_, DIC, DOC and nutrients (PO_4_^3−^, NO_3_^−^, NO_2_^−^, NH_4_^+^) across the sediment-water interface, benthic chambers were deployed over three types of carbonate sediment: (1) sediment covered with BCMs (BCM treatment), (2) sediment initially covered with BCMs, but experimentally removed (CTRL 1 treatment), and (3) sediment without BCMs (CTRL 2 treatment).

We used a modified version of the *in situ* benthic chamber used in Cook et al.^46^ and Huettel and Gust^47^. The benthic chambers consisted of an acrylic cylinder (Ø 190 mm) with a compensator bag for diver-operated time-series sample retrieval (Fig. S1b). The chamber were inserted into the sediment (10–15 cm) and sealed with a lid. Mixing of the overlying water was maintained by a rotating acrylic stirrer disc (10 cm diameter). The stirring speed of the disk was set to a “non-advective mode” at 20 rpm with a reversing rotational direction every 15 s to ensure mixing without creating a pressure gradient^46^. The mixing process was validated by adding a tracer (ink) and following the color visually over time and space prior to the experiments. After addition of the tracer, the stirred chamber was entirely and homogeneously colored within two minutes. The chamber enclosed a seafloor area of 284 cm^2^. BCMs covered ≥90% of the surface area of each benthic chamber for the BCM and CTRL 1 (i.e. before experimental removal) treatments (Fig. S1b). The overlaying water column was 4–6 liters (equivalent to chamber height of 10–15 cm).

Incubations were performed day- (start: 10:30 AM ±30 mins) and night-time (start: 08:30 PM ±30 min) for a duration of 6 h each. Water samples (180 ml) were slowly withdrawn over a period of 5 min from the overlying water of the chambers through a stopcock at the start (T0), after 3 h (T3) and after 6 h (T6). The replacement of the sample volume was ensured through a volume compensator attached to the chamber (Fig. S1b).

The chamber set up consisted of four individual chambers linked to a single battery by 2 m long cables which prevented placing the chambers simultaneously in sediments with BCMs (BCM treatment) and without BCMs (CTRL 2 treatment), but allowed each chamber to be positioned at least 2 m apart. Thus, all four chambers were first deployed on sediment with BCMs (BCM treatment), with day and night incubations performed over two consecutive days (i.e. day 1: day incubations, day 2: night incubations) without moving the chambers. On day 3, the chambers were left in place, their lids were opened and all visible mats were removed by hand picking for a few minutes at least 18 h before the start of the CTRL1 incubations for inducing equilibrium of sediment. The mats were not embedded in the sediment. They formed a unit of dense intertwined filaments which could be easily detached from the carbonate sediment without affecting the sediment surface layer. After removal, sediment with BCMs removed had the same visual appearance as surrounding sediment without BCMs. Another batch of day and night incubations were run on day 4 and 5 (CTRL 1 treatment). In between running the BCM and CTRL 1 incubations, the chambers were left opened to allow water exchange. The chambers were then moved to an area free of BCMs to run the CTRL 2 day and night incubations. The same procedure was repeated twice using new patches (total deployment area: ca. 500 m^2^), followed by an additional batch of BCM chambers, resulting in 12 replicates for the BCM treatment, and 8 replicates for both CTRL 1 and CTRL 2 treatments for each day and night incubation. Light and temperature was monitored during the experiment using loggers (Hobbo Pendant, Onset).

### Sample processing

Oxygen concentrations were measured after retrieval of samples on land at *in situ* temperature with an oxygen optode (Hach HQ10 + LDO). Salinity was measured with a refractometer to check for groundwater seepage which, if present, would be expected to lower salinity. For DIC analyses, 6 ml of each sample was transferred into gas tight glass tubes (Exetainers) without headspace, fixed with mercury chloride, and stored in the dark at 4°C. DIC concentrations were measured with the flow injection method (conductivity detector: VWR scientific model 1054) according to Hall & Aller^48^.

Samples for DOC (40 ml) were filtered (<20 kPa Hg suction pressure) over a 0.2 μm polycarbonate filter (Whatman, 25 mm). Prior to filtration, filters, glassware and pipette tips were rinsed three times with acid (10 ml 0.4 M HCl) and twice with sample water (10 ml). Afterwards 20 ml of the sample water was filtered, each filtrate containing DOC was transferred to a pre-combusted (4 h at 450°C) glass ampoule and sealed immediately after acidification with 6–7 drops of concentrated HCl (38%) to remove inorganic C and stored at 4°C until analysis. There were not enough glass ampoules to measure DOC in the three experimental treatments so CTRL 2 was excluded. DOC concentrations were measured using a total organic C analyzer (TOC-VCPN; Shimadzu) according to Ogawa et al.^49^. The instrument was calibrated with a standard addition curve of Potassium Phthalate (0; 25; 50; 100; 200 μmol C l^−1^). A consensus reference materials provided by Hansell and Chen of the University of Miami (Batch 12, 2012; 41–44 μmol C l^−1^) was used as positive control. Concentrations measured for the entire batch gave an average value of 45 (±2) μmol C l^−1^. Average analytical error of the instrument was <3% (5–7 injections per sample).

Samples for nutrients (50 ml) were immediately filtered with 0.22 μm syringe filters (Minisart® NML sterile Syringe Filters 16534, Hydrophilic), stored in 6 ml Pony vials and frozen (−20°C). Nutrients were also analyzed at NIOZ, Texel, using continuous flow analysis via a Quatro auto-analyzer (Seal Analytical, UK) following the methodologies of Grasshoff et al.^50^ for NO_3_^−^ and NO_2_^−^, Helder & De Vries^51^ for NH_4_^+^ and Murphy & Riley^52^ for PO_4_^3−^.

### Flux and carbon budget

Fluxes of O_2_, DIC, DOC and nutrients were calculated from the linear regression of the respective concentration versus time^53^:
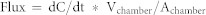
where dC/dt is the change of the concentration over the incubation time, V_chamber_ is the volume of enclosed bottom water, and A_chamber_ is the surface area enclosed by the chamber. Positive fluxes show a release of the solute across the sediment-water interface into the bottom water, while negative fluxes indicate an uptake of the solute. Error estimates caused by water efflux through the sediment were calculated for all chambers using maximal and minimal values for each treatment. Flux data were tested by one-way ANOVA with experimental treatment (i.e. BCM vs CTRL 1 vs CTRL 2) as fixed factor for each day and night period, followed by Scheffe posthoc tests.

Carbon budget calculations were based on the assumption that for each mole of oxygen produced/respired, one mole of carbon is fixed/respired (1:1). Oxygen flux data were thus used as a base for the calculations. Carbon budgets were estimated for sediments with and without BCMs using data from the BCM and CTRL 1 treatments, respectively. O_2_ fluxes during the day were used as net production rates (NP). Carbon budgets were then calculated by subtracting from NP all carbon losses by respiration in the night and DOC releases/uptakes. Standard deviation (SD) of each carbon budget was calculated by taking the square root of the sum of all SD each to the power of two used in the calculation.

### Reef scale DOC calculations

To compare DOC release rates of BCMs with other reported rates, we compiled data on benthic primary producers of coral reefs from the literature (Table 2). To estimate the contribution of BCMs to the DOC pool in the water column at the reef scale, percent cover of major benthic groups (BCMs, macroalgae, turf, corals and sediment not covered by BCMs) were determined from 20 quadrats of 4 m^2^ (2 × 2 m), which were haphazardly placed at 7 m depth at Pest Bay. Each quadrat was photographed on November 2011 using a series of four overlapping photographs (ca. 1.5 m^2^ each) which were subsequently assembled to make one overview photograph. Each overview photograph was analysed using the program Coral Point Count with Excel Extensions (CPCe) using 120 points^54^. DOC release/uptake rates for BCM and sediment were calculated using data from the BCM and CTRL 1 treatments, respectively. DOC release/uptake rates for macroalgae (*Dictyota* sp.), corals (*Madracis* sp.) and turf (daytime only) were taken from Mueller et al.^16^. Mueller et al.^16^ determined DOC release in *ex situ* incubations in April 2011 (daytime incubations) and between May and July 2010 (dark incubations) using samples collected at 8 m depth near the Carmabi biological research station ca. 5 km away from our study site (Fig. S1a; 12°7′18.06″N, 68°58′10.59″W). Night DOC release for turf was not available in their study. Thus data were taken from Haas et al.^19^, which conducted nighttime *ex situ* incubations using turf algae collected at 2–2.5 m depth in Moorea, French Polynesia. Individual DOC release/uptake rates over a diel cycle were multiplied by the cover of the major benthic components at our study site to obtain their respective contribution to the DOC pool at reef scale.

### *In situ* sediment oxygen profiles

To document the mat activity, vertical profiles of dissolved oxygen were measured over a 24 h cycle at 40 min intervals in the center and outside of five BCM patches at Pest Bay using an *in situ* diver operated microsensor system^55^. Profiles were measured in 200 μm steps until anoxic sediments (i.e. consistently low values) were detected. A 2-point calibration was performed using the constant signal in the well-mixed overlying water ~50 cm above the seabed (assuming the overlying water was 100% saturated with oxygen, which was confirmed by the constant sensor reading at the start of the each profile and occasionally checked by optode measurements) and in the deeper anoxic sediment. Analysis of the profiles was done using custom-made programs MPR-plotter and L@MP.

## Supplementary Material

Supplementary Information Figure S1

## Figures and Tables

**Table 1:** Estimated fluxes (mmol C m^−2^ h^−1^), light (μmol photons m^−2^ s^−1^) and temperature (°C) for sediment with BCMs (BCM), after experimental removal (CTRL 1), and for sediment without BCM (CTRL 2). Flux calculations are based on 6 h except for O_2_ and DIC due to flux changes in the last hours caused by high concentrations. DOC fluxes are not available for CTRL 2 due to a shortage of sample containers. Differences among treatments from each day and night period were analysed by one-way ANOVA (Significance <0.05, ns = non significant). Significance column indicates homogeneous subgroups by posthoc Scheffé tests. n.s. = not significant. na = data not available

Treatment:	BCM			CTRL 1			CTRL 2			
	Mean		SD	Mean		SD	Mean		SD	Significance
**DAY**										
O_2_	8.77	±	0.86	0.99	±	0.41	1.29	±	0.43	BCM > CTRL1 = CTRL2
DIC	−12.10	±	1.16	−1.94	±	1.26	−1.88	±	1.56	BCM > CTRL1 = CTRL2
DOC	1.36	±	1.21	−0.85	±	0.67	na	±	na	BCM > CTRL1
Light	193.8	±	49.0	245.6	±	16.2	163.6	±	5.7	n.s.
Temperature	29.9	±	0.3	30.1	±	0.2	29.7	±	0.3	BCM = CTRL1 > CTRL2
Error (%)^a^	4.4–8.7	±	2.9–5.8	5.2–10.5	±	2.6–5.2	4.5–9.0	±	3.3–6.6	
**NIGHT**										
O_2_	−3.25	±	0.81	−1.14	±	0.73	−1.39	±	0.36	BCM > CTRL1 = CTRL2
DIC	8.75	±	2.60	2.24	±	1.16	2.44	±	2.30	BCM > CTRL1 = CTRL2
DOC	3.52	±	2.03	−0.64	±	0.69	na	±	na	BCM > CTRL1
Temperature	29.3	±	0.3	29.3	±	0.3	28.9	±	0.2	BCM = CTRL1 > CTRL2
Error (%)^a^	4.5–9.0	±	3.4–6.7	6.6–13.1	±	3.3–6.6	4.6–9.2	±	3.8–7.6	

^a^Error estimates for flux data.

**Table 2:** Reported DOC releases (mmol C m^−2^ h^−1^) of different primary producers on coral reefs

Group	Division	Species	DOC release^a^		Reference
			Day	Night/Dark	
Macroalgae	Chlorophyta	*Avrainvillea* sp.	−0.50	n.a.	[34]
		*Caulerpa* sp.	0.56 to 1.11	0.05	[27]
		*Cladophora* sp.	2.02	0.05	[16]
		*Enteromorpha* sp.	0.14	n.a.	[27]
		*Halimeda opuntia*	0.21	n.a.	[28]
		*Halimeda opuntia*	2.85	n.a.	[16]
		*Halimeda* sp.	−0.07	n.a.	[34]
		*Penicillus* sp.	0.25	n.a.	[34]
		*Rhipocephalus* sp.	1.01	n.a.	[34]
		*Ulva* sp.	0.28	n.a.	[27]
		**Range:**	**−0.50 to 1.11**		
	Phaeophyta	*Dictyota ceylanica*	0.48	0.16	[19]
		*Dictyota menstrualis*	0.49	−0.01	[16]
		*Hydroclathrus* sp.	0.41	n.a.	[27]
		*Lobophora variegata*	0.49	−0.01	[16]
		*Lobophora* sp.	0.40	n.a.	[27]
		*Lobophora* sp.	0.85	n.a.	[34]
		*Sargassum* sp.	0.47	n.a.	[27]
		*Turbinaria ornata*	0.49	n.a.	[28]
		**Range:**	**0.40 to 0.85**		
	Rhodophyta	*Amansia rhodantha*	0.80	n.a.	[28]
		*Hydrolithon reinboldii*	0.47	n.a.	[28]
		*Hydrolithon reinboldii*	0.24	0.04	[19]
		*Liagora* sp.	0.41	n.a.	[27]
		*Lithophyllum congestum*	5.35	n.a.	[16]
		*Peyssonnelia* sp.	0.24 to 2.96	n.a.	[27]
		**Range:**	**0.24 to 5.35**		
Turf	Consortia	turf algae	0.52 to 5.53	n.a.	[27]
		turf algae	1.40	n.a.	[28]
		turf algae	1.08	n.a.	[16]
		turf algae	0.46	0.11	[19]
		**Range:**	**0.52 to 5.53**		
Scleractinian	Scleractinia	*Acropora formosa*	1.25	n.a.	[56]
corals		*Acropora nobilis*	2.22	n.a.	[21]
		*Acropora pulchra*	0.37	n.a.	[57]
		*Acropora* sp.	2.56	−0.15	[22]
		*Fungia* sp.	−1.18	n.a.	[22]
		*Goniastrea* sp.	1.83	n.a.	[22]
		*Madracis mirabilis*	−0.87 to 0.91	−0.27	[16]
		*Manicina* sp.	−13.03	n.a.	[34]
		*Millepora* sp.	0.77	n.a.	[22]
		*Montipora digitata*	0.09	n.a.	[57]
		*Orbicella annularis*	0.15	3.01	[16]
		*Pocillopora damicornis*	0.07	0.07	[19]
		*Pocillopora* sp.	−21.93	n.a.	[22]
		*Porites lobata*	0.18	n.a.	[28]
		*Porites* sp.	3.17	n.a.	[34]
		*Stylophora* sp.	−1.17	n.a.	[22]
		**Range:**	**−21.93 to 3.17**		

^a^DOC release rates are measured under different experimental conditions, such as various light intensities, and do not picture the rates of an entire day, only the release per hour during the short *in vivo* incubations at daytime.

**Table 3:** a) Estimated DOC release on the reef flat at Pest Bay. b) Scenario without BCMs. BCMs were replaced with BCM-free sediment

	DOC release (mmol C m^−2^ h^−1^)			Benthic cover (%)	Reef DOC release (mmol C m^−2^ reef h^−1^)	References
	Day	Night	24 hrs^a^		24 hrs^a^	
a)						
BCMs	1.36	3.52	2.44	24	0.59	Present study
Macroalgae (*Dictyota*)	0.49	−0.01	0.24	17	0.04	[16]
Turf	1.08	0.11	0.60	19	0.11	Day^16^ Night^19^
Sediment	−0.85	−0.64	−0.75	25	−0.19	Present study
Corals (*Madracis*)	−4.58	−1.42	−3.00	13	−0.39	[16]
Total				98	**0.16**	
b)						
Sediment	−0.85	−0.64	−0.75	24	−0.18	Present study
Macroalgae (*Dictyota*)	0.49	−0.01	0.24	17	0.04	[16]
Turf	1.08	0.11	0.60	19	0.11	Day^16^ Night^19^
Sediment	−0.85	−0.64	−0.75	25	−0.19	Present study
Corals (*Madracis*)	−4.58	−1.42	−3.00	13	−0.39	[16]
Total				98	−**0.60**	

^a^assuming 12 h each for both day and night.
