# Supplementary material for: High dissolved organic carbon release by benthic cyanobacterial mats in a Caribbean reef ecosystem
Source: Sci Rep. 2015 Mar 9;5:8852. doi: 10.1038/srep08852 (PMC4649756; doi:10.1038/srep08852)
Supplement: Supplementary Information — Figure S1 [file srep08852-s1.pdf]

Supplementary Information:

High dissolved organic carbon release by benthic cyanobacterial mats in a Caribbean reef ecosystem

Hannah J. Brocke, Frank Wenzhoefer, Dirk de Beer, Benjamin Mueller, Fleur C. van Duyl, Maggy M. Nugues

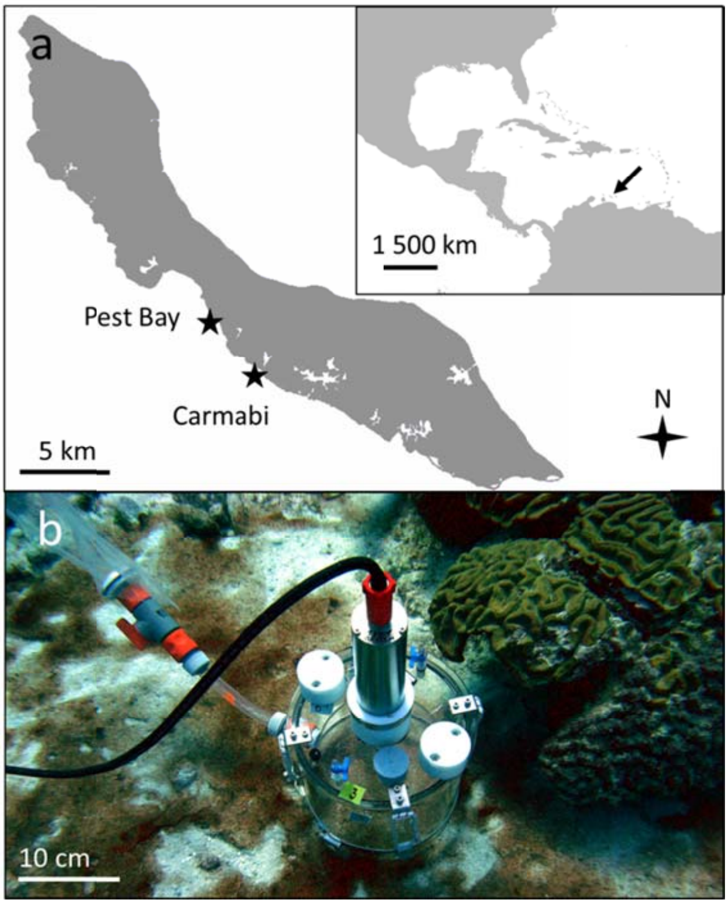

Figure S1. (a) Map of the island Curaçao. Stars display locations of the experimental site Pest Bay and Carmabi. *Inset* shows Central America and the Caribbean Sea. Arrow indicates the location of Curaçao. Maps produced using GeoMapApp (v.3.4.) and GIMP (v.2.8.). **(b) Photo of sediment surface and incubation chamber next to a coral head.** Carbonate sediments are covered by brown-colored BCMs.
